# Modified NiFe_2_O_4_-Supported Graphene Oxide for Effective Urea Electrochemical Oxidation and Water Splitting Applications

**DOI:** 10.3390/molecules29061215

**Published:** 2024-03-08

**Authors:** Fowzia S. Alamro, Shymaa S. Medany, Nada S. Al-Kadhi, Hoda A. Ahmed, Mahmoud A. Hefnawy

**Affiliations:** 1 Department of Chemistry, College of Science, Princess Nourah bint Abdulrahman University, P.O. Box 84428, Riyadh 11671, Saudi Arabia; 2 Chemistry Department, Faculty of Science, Cairo University, Giza 12613, Egypt; 3 Chemistry Department, College of Sciences, Taibah University, Yanbu 46423, Saudi Arabia

**Keywords:** urea electrooxidation, nickel ferrite, water splitting, graphene oxide, fuel cells

## Abstract

The production of green hydrogen using water electrolysis is widely regarded as one of the most promising technologies. On the other hand, the oxygen evolution reaction (OER) is thermodynamically unfavorable and needs significant overpotential to proceed at a sufficient rate. Here, we outline important structural and chemical factors that affect how well a representative nickel ferrite-modified graphene oxide electrocatalyst performs in efficient water splitting applications. The activities of the modified pristine and graphene oxide-supported nickel ferrite were thoroughly characterized in terms of their structural, morphological, and electrochemical properties. This research shows that the NiFe_2_O_4_@GO electrode has an impact on both the urea oxidation reaction (UOR) and water splitting applications. NiFe_2_O_4_@GO was observed to have a current density of 26.6 mA cm^−2^ in 1.0 M urea and 1.0 M KOH at a scan rate of 20 mV s^−1^. The Tafel slope provided for UOR was 39 mV dec^−1^, whereas the GC/NiFe_2_O_4_@GO electrode reached a current of 10 mA cm^−2^ at potentials of +1.5 and −0.21 V (vs. RHE) for the OER and hydrogen evolution reaction (HER), respectively. Furthermore, charge transfer resistances were estimated for OER and HER as 133 and 347 Ω cm^2^, respectively.

## 1. Introduction

Urea electrooxidation is an important technology in the field of electrochemical energy conversion. It enables chemists to convert urea, a renewable resource, into useful forms of energy. This can be achieved through electrochemical oxidation, which involves the application of an electrical current to a solution containing urea molecules [1,2,3,4,5]. 

Low-cost Ni-based catalysts, such as nickel hydroxides, nickel alloys, nickel chalcogen, and nickel phosphide, have been developed for various applications, including hydrogen production, CO_2_ reduction, and water splitting [6,7]. These Ni-based catalysts have demonstrated promising activity, stability, and selectivity results, making them a potential substitute for expensive noble metal catalysts. It has been discovered that nickel hydroxides (Ni(OH)_2_) are more promising candidates for UOR [7,8,9].

Researchers have recently created various green energy systems to efficiently produce H_2_, including two-electrode electrolysis of water, water splitting using a photoelectrode device, solar cells, thermoelectric devices, triboelectric nanogenerator, as well as other devices like pyroelectric and the water–gas shift (WGS) reaction. These green energy technologies may efficiently facilitate water splitting for H_2_ generation [10,11].

The hydrogen evolution reaction (HER) is a crucial element in water splitting devices and significantly hinders energy efficiency because of slow reaction kinetics [12,13,14,15]. The efficiency of the hydrogen evolution reaction is mostly influenced by the catalyst’s activity. Utilizing renewable energy sources like solar and water energy for electrochemical water splitting is a sustainable method to generate green hydrogen. Commercial Pt/C is the most effective catalyst for the hydrogen evolution process (HER) (2H+ + 2e−→H2) [16,17]. However, its high cost and limited availability have hindered its widespread use in industrial production. Many research have focused on creating a novel catalyst for the hydrogen evolution reaction using non-precious metals that are common on Earth [18,19].

The oxygen evolution reaction (OER) is a four-electron process. Despite the use of commercial OER catalysts like RuO_2_ and IrO_2_ made of precious metals, their scarcity and high cost have hindered their commercial use [20,21]. Research efforts are now concentrating more on noble metal-free catalysts, including transition metal-based oxides, phosphates, and hydroxides [22,23]. Currently, enhancing the efficiency of OER electrocatalysis includes using metal doping to modify the electrical properties of the reactants. Transition metal hydroxides, especially those involving oxygen, are being considered as a viable alternative for electrocatalysts because of their cost effectiveness, abundant availability, environmental friendliness, unique atomic structure, and strong catalytic capabilities [24,25]. Several research investigations have shown that FeNi-based materials have outstanding catalytic performance, making them attractive catalysts for enhancing OER activity [26,27,28].

Spinel oxides with a basic structure of AB_2_O_4_ (where A and B are metallic cations) are exceptionally chemically and thermally robust substances suited for various catalytic purposes. The most attractive anodic materials for electrochemical applications are 3d transition metal oxides with a spinel phase [29,30,31,32].

One significant member of the graphene family is graphene oxide (GO). It has several oxygen-containing functional groups. Additionally, despite having -COOH and COH groups at the ends, the defective GO sheets have many COH and CO-C (epoxide) groups [33,34]. These groups make GO hydrophilic by facilitating easy solvent dispersion, resulting in long-term stability [35,36]. GO’s increased functional sites make it a prospective modifying candidate for the attachment of a range of molecules to the surface due to its special and beneficial characteristics, such as its extended surface area, low-cost production, and ease of preparation of dispersions in aqueous media. Due to its special properties, GO has also demonstrated promising results in several applications, including electrochemical sensors, energy storage, fuel cells, and solar cells [37,38,39,40,41,42]. 

Graphene oxide and nickel ferrite were combined to improve urea electrooxidation. Thus, comparative studies were investigated between nickel oxide, pristine nickel ferrite, and modified nickel ferrite. Alternatively, various electrochemical methods were used to describe the activity of the changed surfaces. Some kinetic parameters were calculated to identify the best surface for urea electrooxidation. Additionally, the electrode’s performance in applications for water splitting was examined. In alkaline media, the development of hydrogen and oxygen was studied. 

## 2. Results and Discussion

### 2.1. Material Characterization

**Figure 1:**
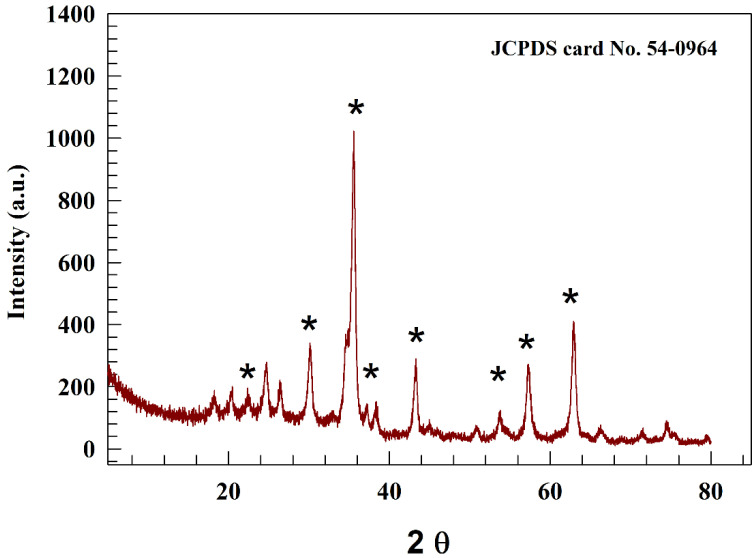
Representation of XRD of NiF_2_O_4_@GO. The * represents the peaks in reference card that reported in the manuscript (JCPDS card No. 54-0964).

The chemical structure of the produced NiFe_2_O_4_ was studied using the powder X-ray diffraction method. Figure 1 displays the XRD chart of NiFe_2_O_4_. Based on reference card JCPDS No.54-0964, NiFe_2_O_4_ exhibited seven distinct peaks at certain angles, namely 2θ = 22.5, 30.2, 35.3, 36.4, 43.2, 53.7, 57.4, and 63.2°, which corresponded to miller indices (111), (220), (311), (222), (400), (422), (511), and (440), respectively. The crystal system of NiFe_2_O_4_ was believed to be cubic, with a crystal point group of m$\bar{3}$m. 

**Figure 2:**
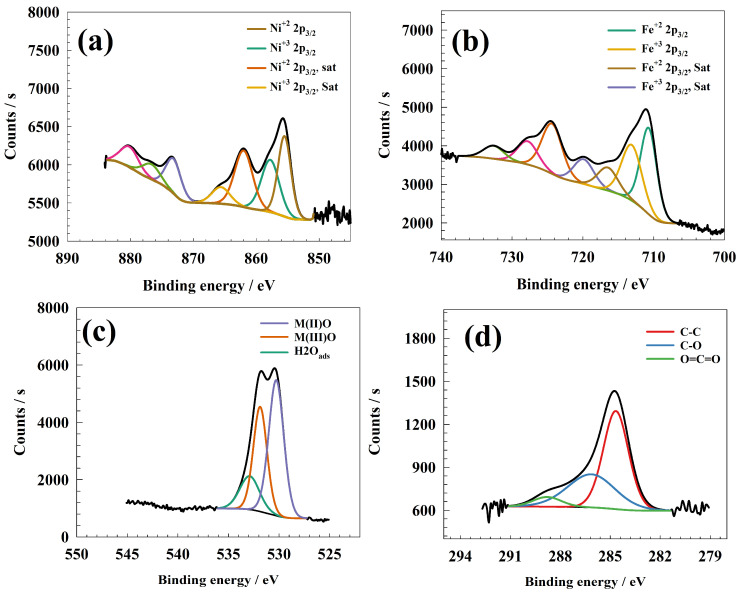
Representation of XPS fitting data of (**a**) Ni2p, (**b**) Fe2p, (**c**) O1s, and (**d**) C1s.

X-ray photon spectroscopy was also used to discover the oxidation states and types of bonds between atoms. The XPS spectra of NiFe_2_O_4_ elements are shown in Figure 2a–d. Ni2p spectra had distinctive peaks at 855.5 and 857.76 eV, which corresponded to the 2p3/2 Ni^2+^ and Ni^3+^ peaks, respectively, as shown in Figure 2a. Meanwhile, the satellite of Ni 2p3/2 caused peaks to be observed at 862.1 and 865.62 eV [43]. Ni 2p1/2 and its satellites were also responsible for the peaks observed at 873.15, 876.67, and 880.14 eV. The XPS spectrum of Fe’s 2p core level is shown in Figure 2b. The spectrum revealed Fe 2p signals attributed to Fe 2p3/2 at 710.69 and 713.08 eV. Peaks for 2p3/2 satellites were also observed at 716.47 and 719.88 eV [44]. Peaks at binding energies of 724.34, 727.79, and 732.61 eV were attributed to Fe 2p1/2 and its satellite. The peaks at 530.28 and 531.87 eV in the O1s spectrum in Figure 2c could be correlated to the M–O of Ni and Fe oxygenated bonds, while the peak located at a binding energy of 532.91 eV was attributed to water molecules adsorbed on the catalyst surface [45]. The C1s spectrum is shown in Figure 2d. C1s showed three distinctive peaks at binding energies of 283.83, 285.84, and 288.21 eV. Additionally, the observed peaks at 283.83 and 285.41 eV were consistent with an extremely thin carbonaceous layer typically present on most air-exposed sample surfaces. The third peak indicated the presence of metal carbonate, which had a binding energy of 288.21 eV.

**Figure 3:**
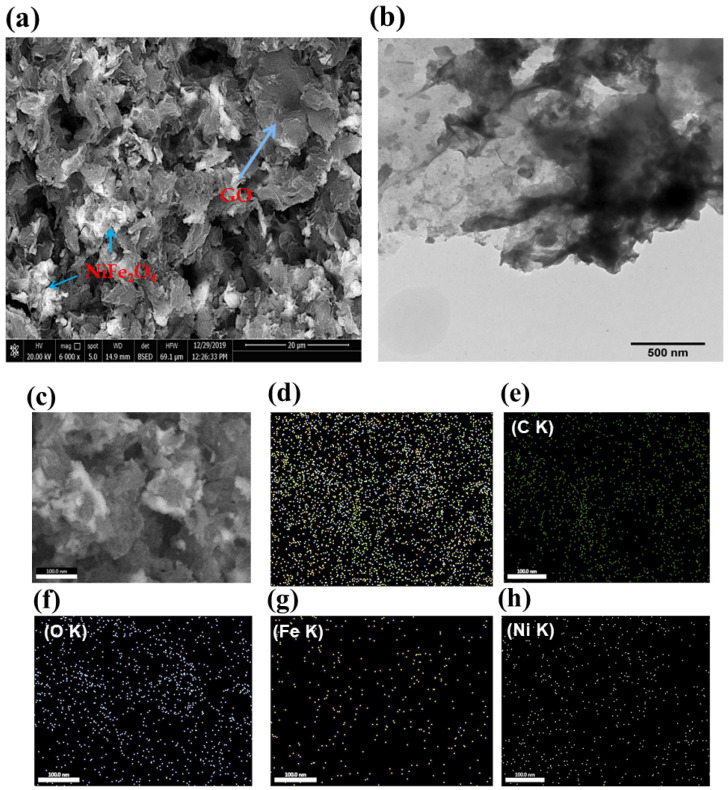

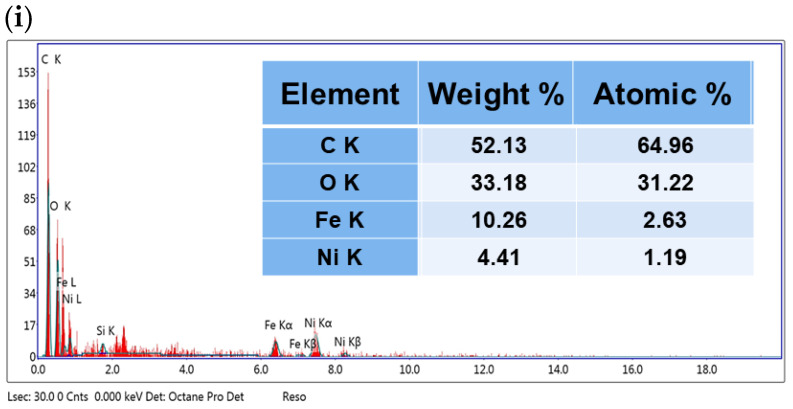
(**a**) SEM and (**b**) TEM of GC/NiFe_2_O_4_@GO sample and corresponding its (**c**–**h**) surface mapping, and (**i**) EDAX.

Scanning electron microscope (SEM) was used to examine the surface morphology of the modified electrode GC/NiFe_2_O_4_@GO electrode. (SEM). Figure 3a shows an SEM image of the surface of the nickel ferrite embedded on the graphene oxide sheets. SEM revealed the presence of catalyst particles with sizes ranging from 40 nm to 90 nm all over the surface. The smaller size structures were aggregated to form the larger size particles. Moreover, the elemental distribution through the electrode surface was revealed in Figure 3c–h. Furthermore, transmitted electron microscope (TEM) was employed to find out the specific distribution of the electrocatalyst on the GO sheet. As represented in Figure 3b, NiFe_2_O_4_ crystal observed on the top of GO sheets confirmed the assumption of nickel ferrite’s surface stability on GO due to oxygenated functional groups. By the XRD analysis results, which confirmed the presence of NiFe_2_O_4_ as the primary phase in the catalyst structure, the EDAX measurement of Figure 3i demonstrated the presence of Ni, Fe, and O with the expected ratio of 1:2:4. The catalyst’s elemental surface mapping revealed that Ni and Fe were evenly distributed across the electrode’s surface.

### 2.2. Electrochemical Urea Oxidation

**Figure 4:**
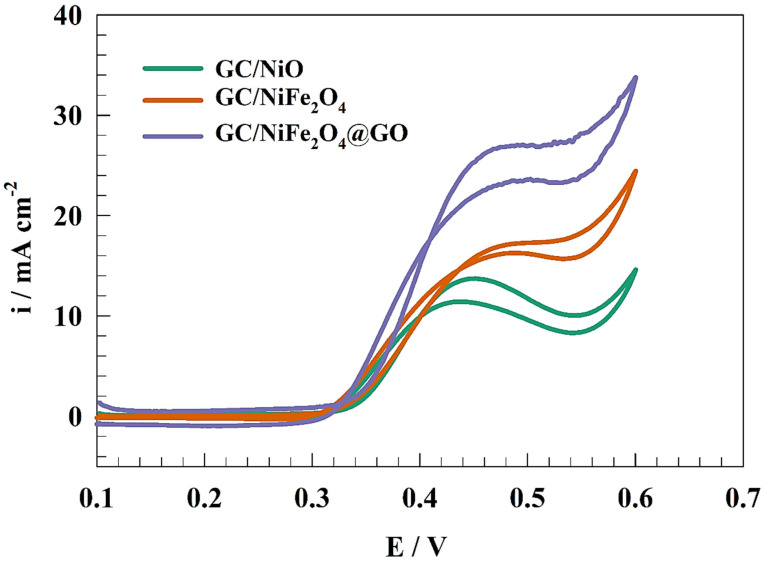
CVs of the different modified electrodes in 1.0 M urea + 1.0 M KOH at 20 mV $s^{-}$^1^.

Cyclic voltammetry, an effective electrochemical method, was employed to examine the redox process that showed the effectiveness of the produced electrocatalysts. Before the measurement, Ni(OH)_2_/NiOOH active species were produced by repeatedly performing 50 CVs in 1.0 M KOH at a scan rate of 50 mVs^−1^. As a result, the thickness of the active species NiOOH increased as the number of successively repeated CVs increased. In the presence of urea, the electrocatalytic activity of the GC/NiO, GC/NiFe_2_O_4_, and GC/NiFe_2_O_4_@GO electrocatalysts were compared for urea electrocatalysis. Thus, different modified electrocatalysts were studied by cyclic voltammetry, as shown in Figure 4, in a solution of 1.0 M urea and 1.0 M KOH at a scan rate of 20 mV s^−1^ with a potential window of 0 to 600 mV. All of the modified GC/NiO, GC/NiFe_2_O_4_, and GC/NiFe_2_O_4_@GO showed two oxidation peaks at a potential range of 420 to 450 mV (vs. Ag/AgCl). The electrode activity was measured as a function of anodic oxidation current.

Consequently, the anodic oxidation current densities were provided as 13.8, 17.2, and 26.6 mA c$m^{-2}$ for GC/NiO, GC/NiFe_2_O_4_, and GC/NiFe_2_O_4_@GO, respectively. Comparing the pristine NiO and nickel ferrite, the higher activity of spinel oxide was attributed to the bimetallic catalyst. On the other hand, grafting the GO with nickel ferrite enhanced the catalytic activity of the electrode toward the UOR. High surface area along with extended functional groups increased the adsorption step for UOR. Also, the oxygenated functional group increased the stability of the electrocatalyst on the electrode surface. 

The presence of Fe along with Ni enhanced the UOR by changing the electronic properties and charge transfer properties of the Ni-based electrodes. The rule of catalyst support is important for an efficient catalysis process. Graphene oxide is considered a carbon-based catalyst with a high surface area. The GO’s functional groups facilitated urea adsorption on the electrode surface. The output anodic current was within the acceptable data published in the literature [46,47]. As a result, Table 1 compares various surfaces for the electrooxidation of urea and shows how different analyte concentrations and scan rates were considered.

**Figure 5:**
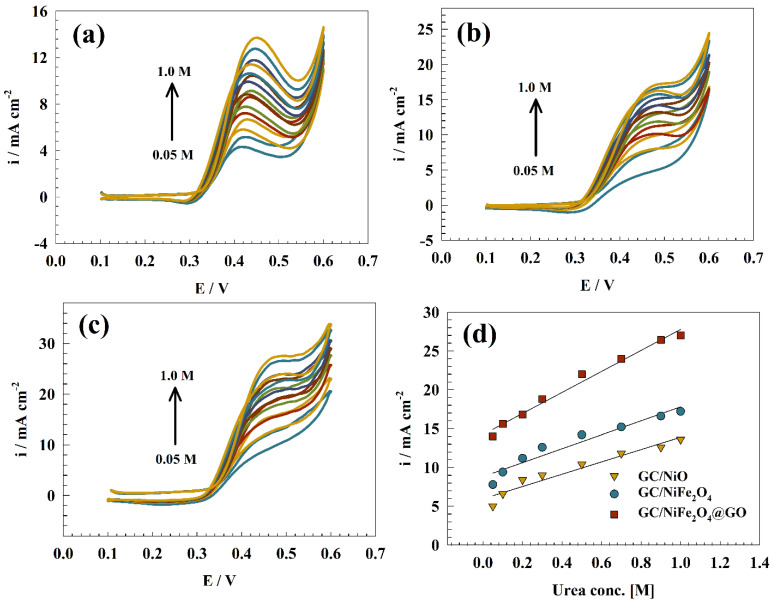
CVs of (**a**) GC/NIO, (**b**) GC/NiFe_2_O_4_, and (**c**) GC/NiFe_2_O_4_@GO at a range of urea concentrations (0.05 to 1.0 M). (**d**) Linear relationship between urea concentration and anodic oxidation current.

The study also evaluated how the GC/NiO, GC/NiFe_2_O_4_, and GC/NiFe_2_O_4_@GO modified electrodes responded electrochemically to changes in fuel concentration. As shown in Figure 5a–c, the effect of varying urea concentration (0.05 to 1.0 M) was studied using a scan rate of 20 mV s^−1^ in a solution containing 1.0 M KOH. The anodic peak current increased along with the concentration of urea. These findings demonstrated the potential of the suggested composite for urea electrooxidation in fuel cells, hydrogen production, and wastewater treatment where urea concentrations vary noticeably. The relationship between the urea concentration and the anodic peak current is shown in Figure 5d. According to the study’s findings, the proposed composite can be a good replacement for urea electrooxidation because of its versatility in how well it operates at various urea concentrations. The anodic current of modified NiFe_2_O_4_@GO composite was compared with other nickel-based electrodes in the literature, as reported in Table 2.

**Figure 6:**
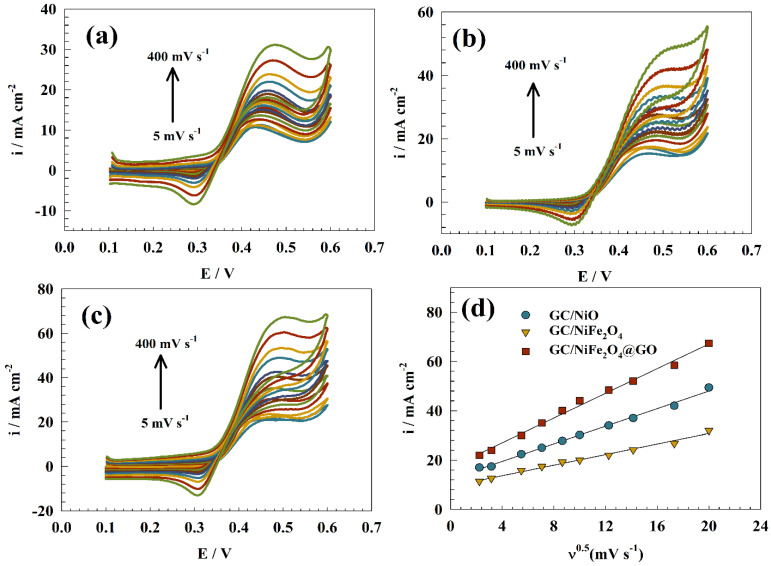
CVs of (**a**) GC/NIO, (**b**) GC/NiFe_2_O_4_, and (**c**) GC/NiFe_2_O_4_@GO at a wide range of scan rates (5 to 200 mV s^−1^) in a solution of 1.0 M urea + 1.0 M KOH. (**d**) Linear relationship between the square root of the scan rate and anodic oxidation current.

As shown in Figure 6a–c, changing the scan rate affected the electrochemical behavior of the GC/NiO, GC/NiFe_2_O_4_, and GC/NiFe_2_O_4_@GO electrodes in 1.0 M urea and 1.0 M KOH ranging from 5 to 200 mV $s^{-1}$. Using the Randles–Sevcik equation (Equation (1)), the relationship between the scan rate’s square root and anodic peak current was plotted to determine the diffusion coefficient. The findings revealed that the oxidation current increased with the scan rate.

Ip = 2.99 × 10^5^ n (1 − α) n_o_A C_o_ D^0.5^ν^0.5^ (1)

where Ip is the maximum oxidation peak current, n is the number of electrons, n_o_ is the number of electrons of the rate-determining step, v is the scan rate, and C_o_ is the urea concentration. The electrode’s surface area was equal to 0.0707 cm^2^, and its diffusion coefficient (D) was in (cm^2^ s^1^). The linear relationship between the current Ip at the anodic peak and the square root (ν) of the sweep rate was used to estimate the diffusion coefficient value, as shown in Figure 6d. As a result, all electrocatalysts were prepared using a diffusion-controlled kinetics process to oxidize urea. The diffusion coefficients were provided as 6.41 × 10^−6^, 4.27 × 10^−5^, and 5.08 × 10^−5^ cm^2^ s^−1^ for GC/NiO, GC/NiFe_2_O_4_, and GC/NiFe_2_O_4_@GO, respectively. The diffusion coefficient value of the GC/NiFe_2_O_4_@GO electrode reflected its good electrocatalytic activity for urea oxidation.

**Figure 7:**
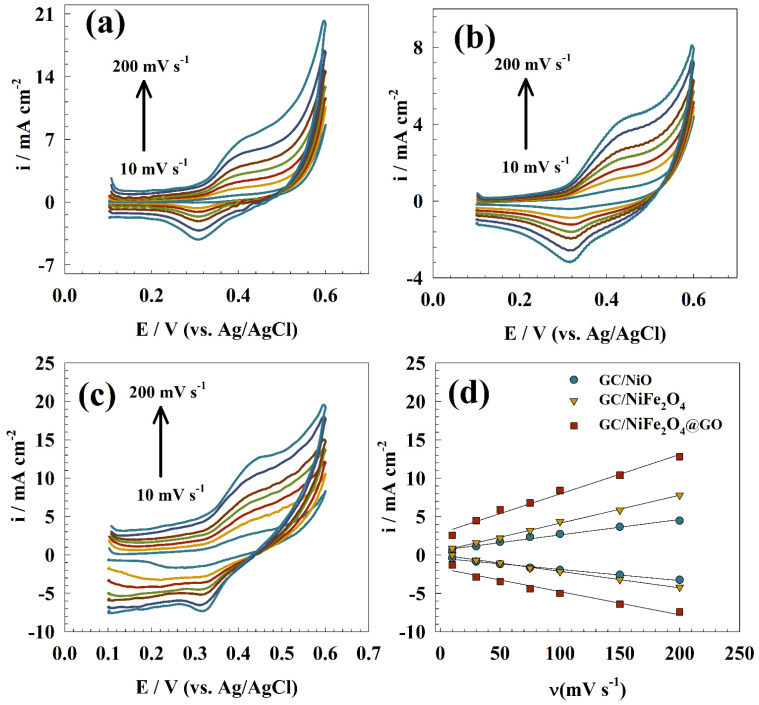
CVs of (**a**) GC/NIO, (**b**) GC/NiFe_2_O_4_, and (**c**) GC/NiFe_2_O_4_@GO in 1.0 M KOH at a wide scan range. (**d**) Linear relationship between scan rate vs. anodic and cathodic currents.

In Figure 7, CVs of the modified electrodes for the Ni(OH)_2_/NiOOH redox pair, namely GC/NiO, GC/NiFe_2_O_4_, and GC/NiFe_2_O_4_@GO, were plotted as a function of scan rate at lower values ranging from 5 to 200 mV $s^{-1}$. Because of the electrochemical activity of retained redox species at the surface of the modified electrocatalysts, straight line relationships were observed. The surface coverage (Γ) values of electrocatalysts could be estimated using Equation (2) [50] and the slope values of the obtained linear plots:

(2) $$Ip=\frac{n^{2}F^{2}}{4RT}\;\nu A\Gamma$$

where Ip is the current of the urea oxidation peak, A is the electrode surface area, and Γ is the redox species surface coverage in mol cm^2^. Table 1 shows the average Γ value for the anodic and cathodic sides of all prepared, GC/NiO, GC/NiFe_2_O_4_, and GC/NiFe_2_O_4_@GO electrocatalysts. Figure 7d shows the linear relationship between the scan rate versus the peak current to calculate the surface coverage. 

Therefore, a modified electrode with graphene oxide GC/NiFe_2_O_4_@GO electrocatalysts had a comparable nickel surface coverage value compared to a pristine NiFe_2_O_4_ electrode. The surface coverage values are reported in Table 1.

**Figure 8:**
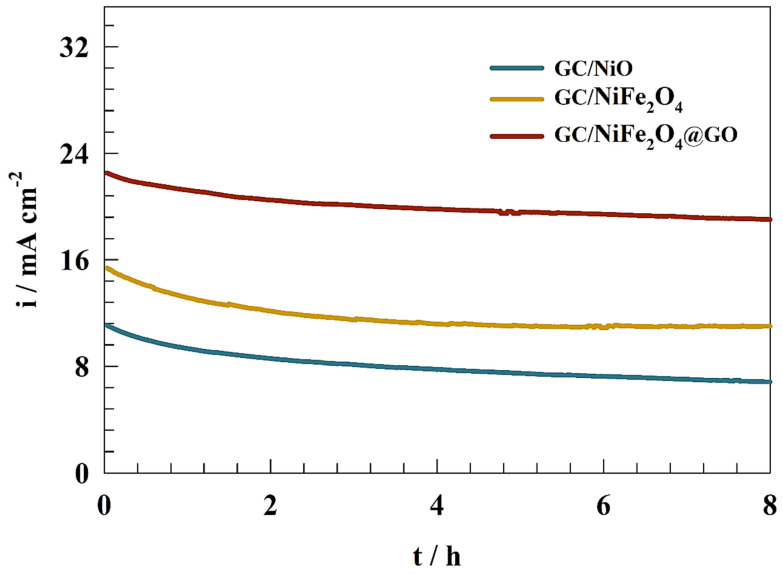
Chronoamperogram of different modified surfaces for urea electrooxidation.

As shown in Figure 8, a chronoamperometry (CA) test was conducted to determine the stability of the synthesized electrocatalysts by applying a constant potential to a 1.0 M KOH and 1.0 M urea solution for 8 h. Thus, the durability of the different electrodes (GC/NiO, GC/NiFe_2_O_4_, and GC/NiFe_2_O_4_@GO) was studied. The electrodes showed high long-term stability toward urea oxidation. The current retention was recorded as 73, 79, and 88% of the initial current values for GC/NiO, GC/NiFe_2_O_4_, and GC/NiFe_2_O_4_@GO, respectively. The presence of an oxygenated functional group in graphene oxide enhanced the oxidation stability by increasing the interaction between the support layer and metal oxides. 

**Figure 9:**
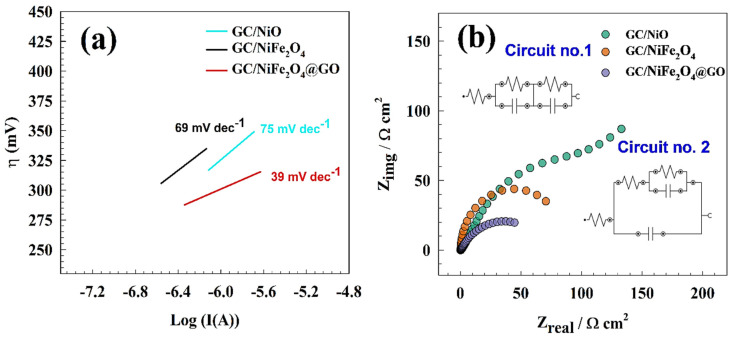
(**a**) Tafel Plot of the modified surfaces. (**b**) Nyquist plot of the modified electrode in 1.0 M urea and 1.0 M KOH, inset figure shows the fitting circuits.

Tafel graphs for urea electrooxidation were generated using the redesigned electrode from quasi-steady state polarization for GC/NiO, GC/NiFe_2_O_4_, and GC/NiFe_2_O_4_@GO in a solution containing 1.0 M urea and 1.0 M KOH. The relationship between the anodic current and overpotential logarithm is shown in Figure 9a. To determine the kinetics of the electrode reactions, Equation (3) is frequently used in electrochemical studies. The Tafel slope value is a crucial parameter that expects the speed of electrons to transfer across the electrode surface. Using the Tafel slope values (75, 69, and 39 mV dec^−1^ for GC/NiO, GC/NiFe_2_O_4_, and GC/NiFe_2_O_4_@GO electrodes, respectively) and the following equation, one can calculate the electron transfer coefficient:

(3) $$Tafel\;Slope=\frac{2.303\;RT}{\left(1-\alpha \right)\;nF}$$

F is Faraday’s constant, T is the absolute temperature, R is the universal gas constant, and n is the number of electrons. Therefore, the calculated electron transfer coefficients were 0.86, 0.85, and 0.74 for GC/NiO, GC/NiFe_2_O_4_, and GC/NiFe_2_O_4_@GO electrodes, respectively. 

Compared to other modified electrodes, this finding suggested that the UOR process is kinetically preferred over the GC/NiFe_2_O_4_@GO electrode. 

For various modified glassy carbon electrodes (GC/NiO, GC/NiFe_2_O_4_, and GC/NiFe_2_O_4_@GO), the electrochemical impedance experiment was conducted using a 1.0 M urea and 1.0 M KOH solution at a constant AC potential of 480 mV. Figure 9b displays the Nyquist plots for several modified surfaces. The Nyquist plots comprised two slightly offset overlapping capacitive semicircles in the high and low-frequency regions. An equivalent circuit inset in Figure 9b was created to fit and simulate the EIS results. The electrode’s outer and inner layers were represented by the R_2_ charge transfer resistance for the outer layer, the R_3_ charge transfer resistance for the inner layer, the C_1_ capacitance for the inner layer, and the C_2_ capacitance for the outer layer. Table 3 provides the values for various EIS parameters. When compared to the other electrodes, it was found that the GC/NiFe_2_O_4_@GO electrode exposed the lowest charge transfer resistance; thus, the quicker the electron transfer occurred during the oxidation process. The electrode with the highest activity displayed a minor diameter, which was correlated with the semi-circuit’s diameter and electrode activity. Therefore, the previously mentioned data successfully demonstrated the activity toward urea electrooxidation based on the EIS results.

### 2.3. Water Splitting Studies

Oxygen evolution significantly converts molecular energy into electrical energy in fuel cells and batteries. It is possible to carry out the anticipated oxygen evolution reaction mechanism via a number of different electrochemical pathways. Usually, two distinct electrochemical mechanisms are used to convert hydrogen oxide to molecular oxygen. The adsorption of ${\left[OH\right]}^{-}$ onto the electrode surface is the first stage, which produces the OH_ads_ species. The second process entails the medium’s interaction with the adsorbed hydroxide group to produce O_ads_. The bound atomic oxygen must be released to produce molecular oxygen. The mechanism of OER has been described as the following:

(4) $$(\mathrm{Ni}-\mathrm{Fe})+OH^{-}↔(\mathrm{Ni}-\mathrm{Fe})OH+e^{-}$$

(5) $$(\mathrm{Ni}-\mathrm{Fe})\mathrm{OH}+OH^{-}↔(\mathrm{Ni}-\mathrm{Fe})O^{-}+H_{2}O$$

(6) $$(Ni-Fe)O^{-}↔(\mathrm{Ni}-\mathrm{Fe})O+e^{-}$$

(7) $$2(\mathrm{Ni}-\mathrm{Fe})O↔2\;(\mathrm{Ni}-\mathrm{Fe})+O_{2}+2e^{-}$$

**Figure 10:**
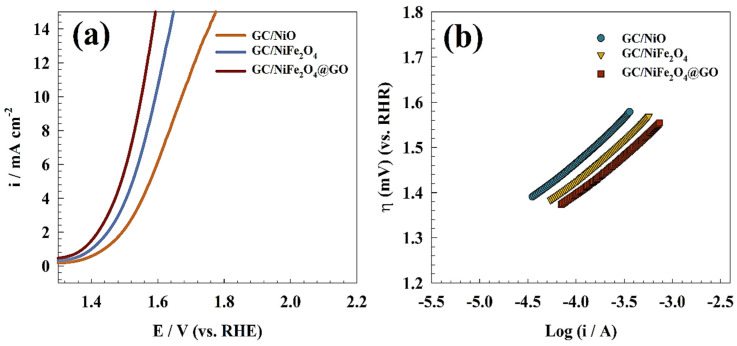
(**a**) Linear sweep voltammetry of the oxygen evolution reaction for the different modified electrodes. (**b**) Tafel slopes of the OER for different surfaces.

Figure 10a shows the OER measured over GC/NiO, GC/NiFe_2_O_4_ and GC/NiFe_2_O_4_@GO in 1.0 M KOH. In comparison to GC/NiO equivalents, high current density for OER was observed in the GC/NiFe_2_O_4_ and GC/NiFe_2_O_4_@GO samples at a lower potential. Additionally, the modified electrodes’ current density reached 10 mA cm^−2^ at potentials of 1.7, 1.6, and 1.5 V (vs. RHE) for GC/NiO, GC/NiFe_2_O_4_, and GC/NiFe_2_O_4_@GO, respectively. Thus, graphene oxide played an essential role in nickel iron composite for enhancing the oxygen evolution ability of the surface. Thus, graphene oxide has been reported in the literature to enhance the OER by shifting the potential to a less positive value [53,54,55]. As depicted in Figure 10b, Tafel plots of the oxygen evolution reaction for GC/NiO, GC/NiFe_2_O_4_, and GC/NiFe_2_O_4_@GO electrodes were examined to determine the influence of electrode composition on the kinetics of electrooxidation. At a scan rate of 1 mV s^−1^, Tafel plots were measured in a 1.0 M KOH solution. Therefore, Tafel slopes were provided as 186, 172 and 162 mV dec^−1^ for GC/NiO, GC/NiFe_2_O_4_ and GC/NiFe_2_O_4_@GO electrodes, respectively. 

**Figure 11:**
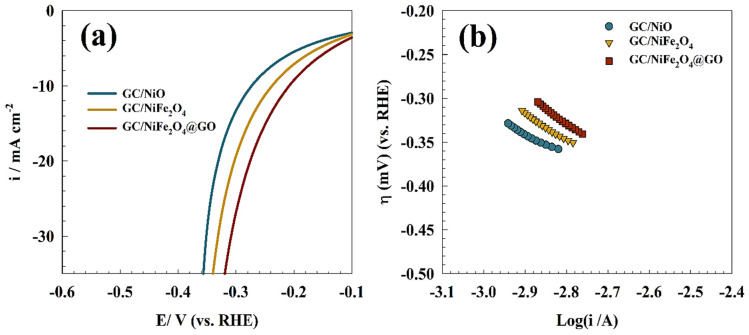
(**a**) Linear sweep voltammetry of the hydrogen evolution reaction for different modified electrodes. (**b**) Tafel slopes of the HER for different surfaces.

Over GC/NiO, GC/NiFe_2_O_4_, and GC/NiFe_2_O_4_@GO modified surfaces, hydrogen evolution processes were investigated by linear sweep voltammetry (Figure 11a) in 1.0 M KOH solution. Thus, the higher catalytic activity of the binary catalyst, like nickel ferrite, was observed by shifting the overpotential of hydrogen evolution toward lower values. Additionally, the electrode activity was enhanced by graphene oxide compared to the pristine nickel ferrite sample owing to the extended surface area and adsorption ability of graphene oxide. The following equation can be used to determine the HER in a very basic environment [56]:

(8) $$2\;H_{2}O+2e^{-}↔\;2\;H_{ads}+2\;OH^{-}\;\;\mathrm{Volmer}\;\mathrm{Step-water}\;\mathrm{dissociation}$$

(9) $$2\;H_{ads}↔H_{2}\;\;\;\;\;\mathrm{Tafel}\;\mathrm{step}$$

(10) $$H_{2}O+H_{ads}+e^{-}↔H_{2}+OH^{-}\;\;\mathrm{Heyrovsky}\;\mathrm{step}$$

The first stage of the HER process involves the adsorption of hydrogen ions (Volmer step) on the electrode surface. Next, a hydrated proton in the medium forms a covalent link with an adsorbed hydrogen atom on the surface (Tafel step) or recombines with two existing hydrogen ions on the surface (Heyrovsky step). To determine whether the first or second phase is the rate-determining phase in hydrogen evolution reactions, Tafel polarization curves using linear sweep voltammetry can be employed. The Tafel diagram of different electrodes, GC/NiO, GC/NiFe_2_O_4_, and GC/NiFe_2_O_4_@GO, is presented in Figure 11b. Consequently, Tafel slopes were calculated as 127, 119, and 104 mV dec^−1^ for GC/NiO, GC/NiFe_2_O_4_ and GC/NiFe_2_O_4_@GO electrodes, respectively. 

**Figure 12:**
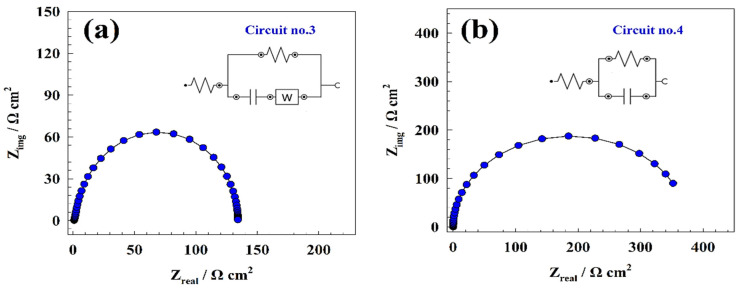
Nyquist plots of the modified GC/ NiFe_2_O_4_@GO electrode for (**a**) OER and (**b**) HER.

At the modified GC/NiFe_2_O_4_@GO electrode, the development of hydrogen and oxygen evolution reactions were examined using electrochemical impedance (EIS). Figure 12a shows the Nyquist curve for the modified electrode GC/NiFe_2_O_4_@GO in the presence of KOH at an AC potential of 1.8 V (vs. RHE). The GC/NiFe_2_O_4_@GO sample demonstrated a semicircle for the oxygen evolution reaction. Thus, the Nyquist plot’s semicircle corresponded to the charge transfer procedure. Using NOVA 2.15 software, oxygen evolution and EIS data were fitted. Solution resistance (Rs) and charge transfer resistance (Rc) are the two resistance elements that express the fitting circuit for the charged electrode. Additionally, charge transfer resistance (Rc) is connected to capacitance (C) and diffusion element (Warburg element), as represented in circuit **no**. **3** (Figure 12a). However, the provided solution and charge transfer resistances were 3.1 and 133.4 Ohm cm^2^, respectively. The HER was utilized by EIS at a constant AC potential of −0.4 V vs. RHE, as shown in Figure 12b. The modified electrode GC/NiFe_2_O_4_@GO exhibited identical semicircle Nyquist plots with varying resistance magnitudes. According to the EIS data, electrochemical production can be approximated as a pure charge transfer process. The electrode showed one charge transfer circuit (see **circuit no**. **4**), which consisted of solution resistance, with one resistance parallel to the capacitor (C) element. However, the resistance of the fitted data showed that solution resistance and charge transfer resistance equaled 7.4 and 347 Ohm cm^2^, respectively. Therefore, the lower estimated resistance reflected the higher activity of the electrode toward oxygen and hydrogen evolution reactions. The fitting circuit parameters for several modified electrodes, GC/NiFe_2_O_4_@GO for hydrogen and oxygen evolution, are shown in Table 4.

## 3. Experimental 

### 3.1. Preparation of Graphene Oxide (GO)

Hummer’s technique was used to create the graphene oxide [57]. In a nutshell, 5% HCl was applied to powdered graphite flakes during the initial stages of preparation. After that, the solution was placed in an ice bath, to which H_2_SO_4_ (98%) and KMnO_4_ were slowly added while being stirred for two hours. Distilled water was mixed into 30% H_2_O_2_ solution to stop the graphite oxidation reaction. Finally, excess metal and salts were removed from the graphene oxide by filtration and several washes with 10%(HCl) solution.

### 3.2. Preparation of Nickel Ferrite (NiFe_2_O_4_)

The sol-gel approach was used to create NiFe_2_O_4_ ferrite [58]. First, suitable quantities of metal nitrates and citric acid were dissolved in a small amount of deionized water. Nitrates had a molar ratio of 1:2 and were in a 1:1 ratio with citric acid. A small amount of polyvinylpyrrolidone (PVP) was then added to the formed solution. The final solution was stirred magnetically for 4 h at room temperature, and any remaining water was then evaporated in a vacuum rotary evaporator at 60 to 80 °C to form a gel. The obtained gel was dried for approximately 10 h in an oven at 80 °C. Finally, brown nickel ferrite powder was produced by a calcinating sample in a furnace at 700 °C for 6 h. 

### 3.3. Electrochemical Measurements

The electrode was prepared by casting 20 mM of catalyst ink on a glassy carbon electrode (GC) with a 3 mm diameter. The catalyst ink was prepared by suspending 20 mg of catalyst (i.e., NiO, NiFe_2_O_4_, or NiFe_2_O_4_@GO) in 1 mL of dimethyl formamide (DMF). Cyclic voltammetry, chronoamperometry, and electrochemical impedance spectroscopy were used in the electrochemical studies. Autolab PGSTAT128N was employed in all electrochemical studies. NOVA 2.15 electrochemistry software was used to analyze the impedance spectrum (Version 2.16). The potentiostat was coupled to a three-electrode cell. The reference electrode was Ag/AgCl/KCl (sat.), and the auxiliary electrode was Pt wire. GC/NiO, GC/NiFe_2_O_4_, and GC/NiFe_2_O_4_@GO electrocatalysts were used as the working electrode. During the electrochemical impedance spectroscopy measurements, a constant AC potential value was adjusted by applying an AC voltage amplitude of 10 mV and a frequency range of $1\times 10^{4}$ Hz to 0.01 Hz. The obtained data were fitted with NOVA 2.15 software using equivalent circuits. All electrochemical experimental studies were carried out at room temperature in deaerated solutions.

The reversible hydrogen electrode (RHE) was used as the reference for the potential:

E_RHE_ = E_Ag/AgCl_ + E°_Ag/AgCl_ + 0.059 pH (11)

The electrochemical experiments were conducted in a solution of KOH containing 1.0 M supporting electrolyte solution. The potential was standardized to a hydrogen electrode that was reversible, as follows:

E°_Ag/AgCl_ = 0.197 V (12)

At pH ~ 14  E_RHE_ = E_Ag/AgCl_ + 1.023 (13)

## 4. Conclusions

The sol-gel technique was used as a practical method for preparing nickel ferrite electrocatalysts. Characterization of the synthesized materials confirmed the synthesis of the nickel ferrite structure. The pristine and graphene oxide-modified nickel ferrite activity was successful used in urea electrooxidation and water splitting applications. Several kinetic parameters were calculated to determine the urea conversion efficiency. The modified GC/NiFe_2_O_4_@GO recorded the highest diffusion coefficient (5.08 × 10^−5^ cm^2^ s^−1^), highest surface coverage (5.49 × 10^−8^ mol cm^−2^), lowest charge transfer resistance (72 Ω cm^2^), and lowest charge transfer coefficient (0.74) among other modified electrode counterparts. The Tafel slopes calculated for GC/NiFe_2_O_4_@GO electrode were 39, 160, and 104 mV dec^−1^ for urea oxidation, the oxygen evolution reaction, and the hydrogen evolution reaction, respectively. 

## Figures and Tables

**Table 1:** Different parameters estimated for the prepared modified electrodes.

	GC/NiO	GC/NiFe_2_O_4_	GC/NiFe_2_O_4_@GO
Anodic oxidation current (mA cm^−2^)	13.8	17.2	26.6
Diffusion coefficient (D)/(cm^2^ s^−1^)	$6.41\times 10^{-6}$	$4.27\times 10^{-5}$	$5.08\times 10^{-5}$
Tafel Slope (mV dec^−1^)	75	69	39
Charge transfer coefficient (α)	0.86	0.85	0.74
Surface coverage(Γ)/(mol cm^−2^)	3.98 × 10^−8^	4.71 × 10^−8^	5.49 × 10^−8^
Onset potential (V)	0.34	0.32	0.33

**Table 2:** Comparison between different surfaces used for UOR.

Electrode	Fuel Concentration (M)	Electrolyte Concentration (M)	Scan Rate (mV s^−1^)	Oxidation Current (mA cm^−2^)	Reference
GC/NiFe_2_O_4_@GO	1.0	1.0	20	26.6	This work
Ni_0.85_Se/rGO	0.5	1.0	50	10	[48]
Ni_0.9_Cu_0.1_	0.3	0.5	20	32	[49]
IN738 supper alloy	1.0	1.0	20	12	[50]
NiO/Fe_3_O_4_@chitosan	0.3	0.5	20	34	[51]
Ni(OH)_2_ meshes	0.3	1.0	50	20	[52]

**Table 3:** EIS fitting parameters for Nyquist plots of different modified surfaces.

Electrode.	R_s_ (Ω)	R_ct_ (Ω)	C_1_ (F)	R_2_ (Ω)	C_2_ (F)
GC/NiO	18.063	592	0.00008864	2575	0.00025987
GC/NiFe_2_O_4_	19.608	37.492	0.00021398	1242	0.00045225
GC/NiFe_2_O_4_@GO	15.131	25.889	0.000292024	1022	0.00065193

**Table 4:** EIS fitting parameters for Nyquist plots of oxygen and hydrogen evolution reactions.

Electrode	R_s_ (Ω cm^2^)	R_ct_ (Ω cm^2^)	C_1_ (F)	W
Oxygen	3.1	133.4	0.0006864	0.00059069
Hydrogen	7.4	347	0.0004173	-

## Data Availability

Data are contained within the article. The datasets used and/or analyzed during the current study are available from the corresponding author on reasonable request.
